# Developing a large-scale dataset of flood fatalities for territories in the Euro-Mediterranean region, FFEM-DB

**DOI:** 10.1038/s41597-022-01273-x

**Published:** 2022-04-12

**Authors:** Katerina Papagiannaki, Olga Petrucci, Michalis Diakakis, Vassiliki Kotroni, Luigi Aceto, Cinzia Bianchi, Rudolf Brázdil, Miquel Grimalt Gelabert, Moshe Inbar, Abdullah Kahraman, Özgenur Kılıç, Astrid Krahn, Heidi Kreibich, Maria Carmen Llasat, Montserrat Llasat-Botija, Neil Macdonald, Mariana Madruga de Brito, Michele Mercuri, Susana Pereira, Jan Řehoř, Joan Rossello Geli, Paola Salvati, Freddy Vinet, José Luis Zêzere

**Affiliations:** 1 Institute for Environmental Research and Sustainable Development, National Observatory of Athens, Athens, 15236 Greece; 2 CNR-IRPI National Research Council-Research Institute for Geo-Hydrological Protection, Rende, 87036 Italy; 3 National and Kapodistrian University of Athens, Faculty of Geology and Geoenvironment, Panepistimioupoli, Zografou, GR15784 Greece; 4 CNR-IRPI National Research Council-Research Institute for Geo-Hydrological Protection, Perugia, 06128 Italy; 5 Department of Geography, Faculty of Science, Masaryk University, Brno, 61137 Czech Republic; 6 Global Change Research Institute, Czech Academy of Sciences, Brno, 60300 Czech Republic; 7 Grup de Climatologia, Hidrologia, Riscs i Paisatge, Universitat Illes Balears, Palma de Mallorca, 07122 Spain; 8 Department of Geography and Environmental Studies, University of Haifa, Haifa, 33000 Israel; 9 School of Engineering, Newcastle University, Newcastle upon Tyne, NE1 7RU United Kingdom; 10 Department of Meteorological Engineering, Faculty of Aeronautics and Astronautics, Samsun University, Ondokuzmayis, Samsun, 55420 Turkey; 11 GFZ German Research Centre for Geosciences, Potsdam, 14473 Germany; 12 Department of Applied Physics, University of Barcelona, Barcelona, 08028 Spain; 13 Department of Geography and Planning, School of Environmental Sciences, Roxby building, University of Liverpool, L69 7ZT Liverpool, United Kingdom; 14 UFZ Helmholtz Centre for Environmental Research, Leipzig, 04318 Germany; 15 National Research Council of Italy - Institute for Agricultural and Forest Systems in the Mediterranean (ISAFOM), Rende, CS Italy; 16 Centre of Geographical Studies, Institute of Geography and Spatial Planning, University of Lisbon, 1600-276 Lisbon, Portugal; 17 Associated Laboratory TERRA, Lisbon, Portugal; 18 University Paul Valéry Montpellier 3, Montpellier, 34090 France

**Keywords:** Natural hazards, Climate-change impacts

## Abstract

This data paper describes the multinational Database of Flood Fatalities from the Euro-Mediterranean region FFEM-DB that hosts data of 2,875 flood fatalities from 12 territories (nine of which represent entire countries) in Europe and the broader Mediterranean region from 1980 to 2020. The FFEM-DB database provides data on fatalities’ profiles, location, and contributing circumstances, allowing researchers and flood risk managers to explore demographic, behavioral, and situational factors, as well as environmental features of flood-related mortality. The standardized data collection and classification methodology enable comparison between regions beyond administrative boundaries. The FFEM-DB is expandable, regularly updated, publicly available, and with anonymized data. The key advantages of the FFEM-DB compared to existing datasets containing flood fatalities are its high level of detail, data accuracy, record completeness, and the large sample size from an extended area.

## Abstract (summary)

**Table:** 

Measurement(s)	flood mortality characteristics • condition (means of transport) • activity • accident place • accident dynamic • death cause • protective behavior • hazardous behavior
Technology Type(s)	data collection
Factor Type(s)	demographics • country • administrative units • NUTS 1–3
Sample Characteristic - Location	territories in the Euro-Mediterranean region

## Background & Summary

Despite significant improvements in managing flood risk and the numerous initiatives governments and institutions undertake, floods threaten human life and health. According to Munich Re^1^, flooding accounted for 40% of all global loss-related natural catastrophes since 1980. In 2020, there were 23% more floods resulting in fatalities and 18% more flood-related deaths compared to the annual average calculated for the previous 20-year period (2000–2019)^2^. In recent decades, Europe has experienced catastrophic floods^3,4^, causing substantial loss of human life^5^, with the river floods of July 2021 resulting in more than 200 fatalities^6^, which demonstrates this problem remains unsolved.

**Fig. 1:**
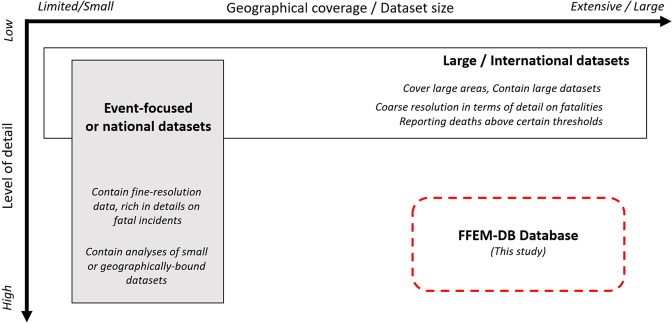
Conceptual model showing the advantages and limitations of existing flood mortality databases and the trade-off between dataset size and detail level, in comparison to the intended position of the FFEM-DB database proposed by the present study.

Insights on how people die from floods usually derive from the study of flood fatality accounts. However, existing studies and databases on flood fatalities (FFs) face important limitations, such as (1) small sample size; (2) narrow geographic extent; (3) low level of detail on FFs; and (4) lack of information concerning the circumstances surrounding fatal incidents (Fig. 1). Regarding the first two limitations, studies tend to focus predominantly on national datasets^7^ or event-focused datasets^8–10^, usually containing a relatively small number of FFs in a specific area. However, examining small samples within narrow geographic boundaries produces results that are hardly transferable to other regions. Such results may be influenced by traditions and cultural factors^11–14^, infrastructure typology^15^, types of environments or settings^16^, housing types^17^, and the population’s quality of training or education. Methodological differences, such as using different systems to classify flood death conditions, are also a major problem for cross-study comparisons (see, for example, Ashley and Ashley^18^ and Fitzgerald *et al*.^19^). A significant challenge for researchers is comparing information for different regions or countries based on common criteria and standards to gain a general, transferable understanding of the drivers of flood mortality.

In addition to these limitations, currently available international databases, such as the Emergency Events Database EM-DAT^20^, provide a useful accounting of fatality numbers but lack details on the circumstances of actual incidents. At the same time, many of them are multihazard-oriented, making the attribution of fatalities to specific hazards, such as flooding, complex, as they often occur in conjunction with other hazards, e.g., wind or landslides. In addition, some international databases include events only if they exceed a minimum threshold of fatalities. Such thresholds lead to a potential miss of fatalities, which, especially within Europe, can be an important portion of the total number of fatalities as a result of a large number of low-mortality events^21^.

To address these gaps, we propose the Database of Flood Fatalities from the Euro-Mediterranean region, FFEM-DB, a multinational database comprising 2,875 FFs from territories in Europe and the Mediterranean region, from 1980 to 2020. FFEM-DB presents an extensive geographical area (covering 12 study areas, nine of which are entire countries) addressing the sample size issue repeatedly acknowledged in the literature^9,22,23^. It provides a high level of detail for each fatality, precise demographic and geographic location data, and details of the circumstances leading to a fatality. Therefore, it enables the comprehensive cross-regional study of FF circumstances, identifying commonalities and differences between particularly vulnerable groups and hazardous situations that lead to fatal accidents, and considering regional socio-economic characteristics. Furthermore, FFEM-DB creates the foundation for studying the association of FF mortality with cross-border variables, such as geomorphological and hydrometeorological features and risk mitigation initiatives and policies. Such cross-regional and cross-border learning can support improved risk communication and better preparedness to help avoid accidents. In this light, FFEM-DB is valuable in evaluating the impact of the EU Flood Directive (2007/60/*EC*) on flood risk management in Europe and relevant to the Sendai Framework for Disaster Risk Reduction target of reducing disaster mortality between 2020–2030.

FFEM-DB brings together the best information available at the regional/national level, ensuring that the data are standardized, verified, and quality controlled. Moreover, FFEM-DB is publicly accessible, and it is scalable as it has been developed with a clearly defined methodology that permits the addition of new regional/national datasets. With these characteristics, the FFEM-DB database is globally unique.

## Methods

### Data sources

The FFEM-DB database draws data from local, high-resolution databases (or datasets) to ensure high accuracy, data quality, and completeness. These databases have been developed and published individually (online-only Table 1) by local research teams or are included here for the first time (e.g., UK) to support mortality studies at national or regional levels. A common denominator of these local databases is the detailed recording of FFs profiles and circumstances of death through multiple sources, namely (1) national authorities, (2) reports from bodies implicated in risk management such as the police and fire department, and (3) local or national media from which detailed information is drawn. Secondary control sources include historical catalogs of damaging flood events/fatalities and scientific publications.

Of the aforementioned data sources, news media were particularly relevant as they are deemed as a reliable source of societal information. They have been previously used to analyze FFs^24–26^, indicate the impact of damaging weather events on a local scale^27–31^, explore the evolution of perception of natural hazards^24,32^, and validate hazard maps^33–35^. The period covered by the FFEM-DB, 1980–2020, can be divided into two time periods based on the availability and ease of access to press information. Roughly, the 1980–2000 period is based on printed archived newspaper material. After 2000, digital newspapers and archives became more abundant, and, most importantly, there is greater access to local newspapers that often provide detailed accounts of particular events. Online-only Table 1 presents the main sources of primary data on fatal flood events and the associated FFs for each study area.

### Data collection and reporting standards

Consistency and accuracy were ensured throughout the data collection process. Securing these criteria concerns two main steps: (1) collecting data from the individual research teams and (2) merging the derived data into FFEM-DB.

Regarding data collection in each study area, various and multiple sources have been used by each independent research team, as shown in the online-only Table 1. All the sources used were specified to ensure high transparency and confidence in the derived information. Despite the variety of sources that may have been used depending on availability, a prerequisite was set that data should be verified by at least two independent sources. We can distinguish different combinations of sources for verifying the data, with the following being the most prevalent among the involved research teams:

- Press and media combined with field research.
- Documentary records, including the press, combined with official authorities’ reports.
- Various media sources using text-mining tools.

To ensure reporting standards were the same for all 12 regions, each research group used a standardized form that enables homogeneity of information imported into the database. This standardized form was developed through a trial period of use by five research groups involved in capturing, designing, and initiating FF data collection^36^ to ensure that it could accommodate their data. The basic form adjustments made during the trial period were as follows:

- Adjust field categorization to cover all distinct sub-cases.
- Introduce new fields and their categorization.
- Revise fields considering the availability and accessibility of the requested information.

The derived data were consistently checked before entering the standard fields of the database. The following were primarily checked concerning the suitability of the reported FFs:

- Each reported FF corresponds to the type of floods the database deals with (see section Data Records).
- Each reported FF is directly associated with rainfall-induced flooding (see section Data Records).

The data collected in the standardized form also included fields for assessing the accuracy of specific information difficult to collect or confirm, namely the approximate hour when the fatality took place and the geographical coordinates. Data was also quality controlled for errors, duplicate entries, and missing data. The identification of duplicate entries was undertaken by computing the Jaccard similarity coefficient^37^. Geographical coordinates were checked in the GIS environment (QGIS 3.10). When needed, coordinates were adjusted based on auxiliary spatial information provided, with either a reverse geocoding process or manual geolocation through Google Maps or OpenStreetMap services. Fatality data were anonymized.

### History and updates of the database

The FFEM-DB is an expandable database that is periodically updated^36,38^. The update occurs on average every two years. It was developed in its original composition in 2017 (MEFF version^36^ with data exclusively from Mediterranean countries or regions for the 1980–2015 period. It included five territories from four countries (France, Greece, Italy, and Spain). In 2019 it evolved to include FFs from new territories in Europe and neighboring non-European countries of the Mediterranean region, covering 1980–2018 (EUFF version^38^). Specifically, the EUFF version also included FFs from the Czech Republic, Israel, Portugal, and Turkey. The FFEM-DB is the latest version, covering a larger area of the Euro-Mediterranean region and a more extended period (1980–2020). Compared to the EUFF version, it also includes FFs from Cyprus, Germany, and the UK. Most importantly, FFEM-DB has been developed into a structured and publicly accessible database, available in 4TU Centre for Research Data^39^. We should note that the standards for collecting, reporting, and controlling data were the same in all database versions.

## Data Records

### Definitions and key concepts

The basic concepts of the FFEM-DB database are defined in the following:

Study period: Currently, FFEM-DB covers 41 years, from 1980 to 2020.

Flood fatality (FF): A person killed by the direct impact of a flood. It encompasses people killed from short-term clinical causes, such as drowning, collapse/heart attack, poly-trauma, poly-trauma & suffocation, hypothermia, suffocation, and electrocution. People missing and presumed dead are included only if more than one source refers to eyewitness testimonies that, for example, the missing person was swept away by a torrent. FFs resulting from storm surges, dam breaks, and accompanying landslides are not included in the FFEM-DB. Additionally, indirect losses associated with long-term health effects are not included.

Fatal flood event (FE): A flash flood or river flood that has caused one or more deaths in a specified region. Flash floods are caused by sudden, short-lived, and usually heavy rainfall over relatively small basin/watershed, while overflowing rivers and streams cause river floods, usually resulting from long-lasting rainfall/snowmelt. Floods caused by the accumulation of rainwater due to lack of drainage, such as urban floods, are also included. The FEs are aggregated at the NUTS 3 spatial level^40^.

### Geographical coverage

**Fig. 2:**
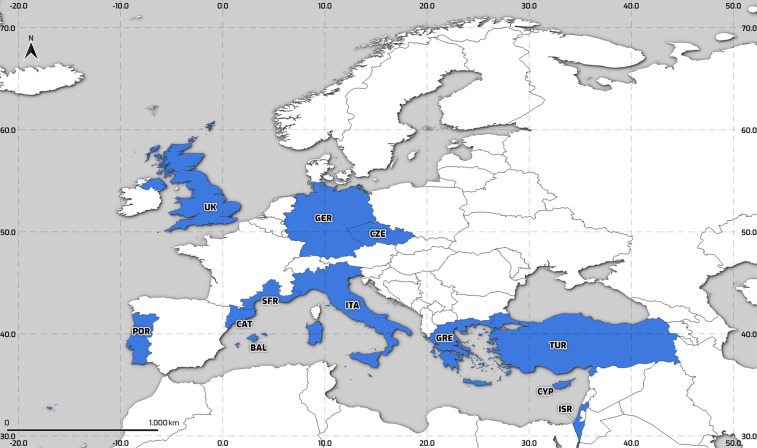
FFEM-DB study areas, in blue. BAL: Balearic Islands; CAT: Catalonia; CYP: Cyprus; CZE: Czech Republic; SFR: Southern France; GER: Germany; GRE: Greece; ISR: Israel; ITA: Italy; POR: Portugal; TUR: Turkey; and UK: United Kingdom.

Nine of the study areas (Fig. 2) represent entire countries: Cyprus (CYP), Czech Republic (CZE), Germany (GER), Greece (GRE), Israel (ISR), Italy (ITA), Portugal (POR), Turkey (TUR), and the United Kingdom (UK). The other three study areas are the Spanish regions of Catalonia (CAT) and Balearic Islands (BAL), as well as the southern French regions bordering the Mediterranean coast (SFR: Languedoc-Roussillon and Provence-Alpes-Cote d’Azur). Table 1 presents information about the area and population of each study area, as well as the number of the representative administrative units at the NUTS 2 level.

**Table 1:** Description of the study areas.

Study area (acronym)	Area (km^2^)	Area (% of total)	NUTS 2 (number of units)	Population (inhabitants)	Population (% of total)	Population density (inh./km^2^)
BAL	4,993	0.2%	1	1,188,220	0.3%	238
CAT	32,113	1.5%	1	7,566,431	2.2%	236
CYP	9,256	0.4%	1	875,899	0.3%	95
CZE	78,873	3.7%	8	10,649,800	3.0%	135
SFR	53,194	2.8%	3	8,343,000	2.4%	157
GER	357,661	16.8%	38	83,019,213	23.7%	232
GRE	131,759	6.2%	13	10,724,599	3.1%	81
ISR	22,159	1.0%	N.A.	9,054,100	2.6%	409
ITA	324,764	15.2%	21	60,359,546	17.2%	186
POR	91,909	4.3%	5	9,779,826	2.8%	106
TUR	780,376	36.6%	26	82,003,882	23.4%	105
UK	244,545	11.5%	41	66,647,112	19.0%	273
Total	2,131,602	100.0%	158	350,211,628	100.0%	—

Notes: Source: https://ec.europa.eu/eurostat/, data 2019. For SFR: French Statistical Service, 2021. For ISR: CBS (2020).

### Database structure and content

Data are stored in a relational MySQL database, using phpMyAdmin administration tool, which consists of three tables: (A) FATALITIES table, (B) LOCATION table, and (C) NUTS 3 table with information on the administrative level, as shown in Table 2. The fields of the table FATALITIES are filled in by selecting from a predefined menu of options, shown in Table 3.

**Table 2:** Structure of the relational FFEM-DB database.

FFEM-DB tables					
FATALITIES		LOCATION		NUTS 3	
FATALITY_ID^A^	Int	FATALITY_ID^A^	Int	NUTS_3_ID^A,B^	Varchar
NUTS_3_ID^B^	Varchar	COUNTRY	Varchar	NUTS_3_NAME	Varchar
DATE	Date	FFEM_STUDY_AREA	Varchar	NUTS_2_ID	Varchar
AGE_STRING	Enum*	STUDY_AREA_ACRONYM	Varchar	NUTS_2_NAME	Varchar
GENDER	Enum*	TERRITORIAL_LV1	Varchar	NUTS_1_ID	Varchar
RESIDENCY	Enum*	TERRITORIAL_LV2	Varchar	NUTS_1_NAME	Varchar
VICTIM_CONDITION	Enum*	TERRITORIAL_LV3	Varchar	NUTS_0_ID	Varchar
VICTIM_ACTIVITY	Enum*	LATITUDE	Decimal	NUTS_0_NAME	Varchar
ACCIDENT_PLACE	Enum*	LONGTITUDE	Decimal	NUTS_3_AREA	Decimal
ACCIDENT_DYNAMIC	Enum*	LOC_ACCURACY	Enum*	NUTS_3_POPULATION	Int
DEATH_CAUSE	Enum*	NUTS_3_ID^B^	Varchar	NUTS_3_POP_DENSITY	Decimal
PROTECTIVE_BEHAVIOR	Enum*			NUTS_3_MALES	Int
HAZARDOUS_BEHAVIOR	Enum*			NUTS_3_FEMALES	Int
				NUTS_3_AGE_0-14_MAL	Int
				NUTS_3_ AGE_0-14_FEM	Int
				NUTS_3_ AGE_15-29_MAL	Int
				NUTS_3_ AGE_15-29_FEM	Int
				NUTS_3_ AGE_30-49_MAL	Int
				NUTS_3_ AGE_30-49_FEM	Int
				NUTS_3_ AGE_50-64_MAL	Int
				NUTS_3_ AGE_50-64_FEM	Int
				NUTS_3_ AGE_OVER64_MAL	Int
				NUTS_3_ AGE_OVER64_FEM	Int
				POP_AGE_NOTE	Varchar

*String objects, ^A^Primary key, ^B^Foreign Key.

**Table 3:** Predefined drop-down menus for the FATALITIES table compilation.

FATALITIES TABLE			
DATE	VICTIM_CONDITION	ACCIDENT_PLACE	PROTECTIVE_BEHAVIOR
Year (yyyy)	By bicycle	Public/private building	Climbing trees
Month (mm)	By boat	Bridge	Driving to avoid danger
Day (dd)	By bus	Campsite/tent	Getting on roof/upper floor
**AGE_STRING**	By car	Riverbed/riverside	Getting out of the car
Child: 0–14 years	By caravan	Tunnel/underpass	Getting out of buildings
Boy/Girl: 15–29 years	By tractor	Countryside	Grabbing onto someone/something
Young adult: 30-49 years	By van	Ford	Moving to a safer place
Adult: 50–64 years	By other	Recreation area	Getting on the car roof
Elderly: >65 years	Laying	Road	**HAZARDOUS_BEHAVIOR**
**GENDER**	Standing	Bungalow	Check damage during the flood
M: Male	**VICTIM_ACTIVITY**	**ACCIDENT_DYNAMICS**	Driving on roads closed by police
F: Female	Traveling	Blocked in a flooded room	Fording rivers
**RESIDENCY**	Recreational activities	Caught in a bridge collapse	Refuse evacuation
Resident	Rescuing someone	Caught in a road collapse	Trying to rescue animals
Not resident	Sleeping	Caught in a building collapse	Refuse warnings
Tourist	Working	Dragged by water/mud	Staying on bridges
	Hunting	Fallen into the river	Staying on river banks
	Fishing	Surrounded by water/mud	Trying to save vehicles
		Hit	Trying to save belongings
		**DEATH_CAUSE**	
		Collapse/hearth attack	
		Drowning	
		Hypothermia	
		Electrocution	
		Poly-trauma	
		Poly-trauma and suffocation	
		Suffocation	

A. FATALITIES table: It contains the date when the fatality occurred, the fatality profile (gender, age, and residency), and circumstances (victim’s condition and activity, the place and dynamics of the accident in terms of the particular circumstances that led to death, clinical cause of death, protective or hazardous behaviors). The ID-Fatality is the primary key connecting this table to the LOCATION table, while NUTS_3_ID works as a foreign key connecting to the table NUTS 3.

B. LOCATION table: It contains administrative and geographical information on where the fatality occurred (country name and acronym, territorial levels from 1 to 3 according to the country administrative subdivisions, latitude and longitude, the accuracy of location). The accuracy of geographical coordinates is considered high if the place of death is precise. Otherwise, it is considered low, and the coordinates correspond to the center of the relevant smaller known administrative unit, e.g., at the territorial_LV3 level.

C. NUTS 3 table: This table allows the downscaling of the location of death from the NUTS 0 (country level) to NUTS 3 level. Geographical and demographic information (area, population, population density, population by gender, and age category) on the NUTS 3 level is also provided^40,41^.

The predefined categories in the fields of the FATALITIES table resulted from extensive research in the existing literature. Previous works have highlighted among FFs the role of demographics^12,42^ and victim activity^22^, infrastructure, the use of vehicles^23,43,44^ and vehicle types^15^, the victim’s residence^42^, and the cause of death^22^. In addition, previous studies have shown the influence of environmental factors^16,45,46^ and the victim’s hazardous behavior^47,48^.

### Spatial data visualization

**Fig. 3:**
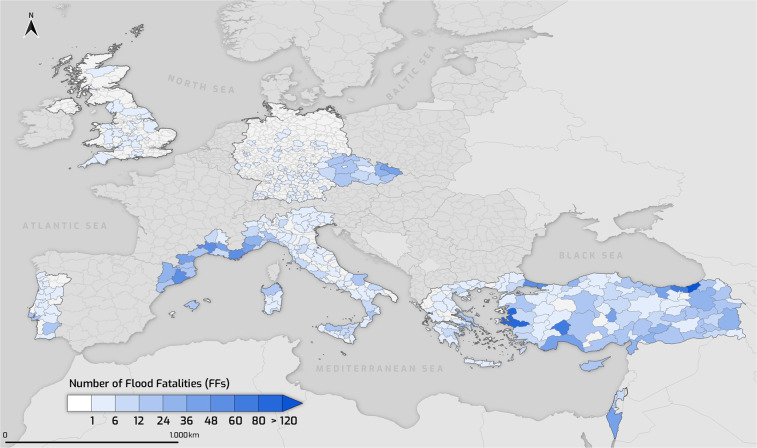
Flood fatalities (FFs) at the NUTS 3 level across the FFEM-DB study areas.

Figure 3 shows the number of FFs at the NUTS 3 level for the examined period. The geographical distribution can indicate various environmental, climatic, and societal factors of vulnerability to flooding. Indeed, analyses published on a previous version of the database^36^ present handy conclusions on the role of the geographical location and demographic features on flood mortality across the studied areas.

## Technical Validation

### Evaluation of completeness and coverage indicators

Based on several indicators, the database is evaluated regarding data completeness and coverage of FFs. The evaluation is undertaken internally, i.e., by evaluating the completeness of the data of each field and study area, and the evolution of completeness within the examined period, as well as externally, by comparing cumulative data against external sources.

Internal evaluation is intended to measure data completeness at various levels and dimensions, to indicate interannual changes, differences between the study areas, and parameters associated with low data availability.

### Field completeness

Table 4 shows the percentages of missing data for each field of the FATALITIES table per study area. The following fields were excluded from the evaluation: (1) the date, which is 100% complete as it is a mandatory field, and (2) the fields of protective and hazardous behaviors, as this information is only available in cases where someone witnessed the accident. Therefore, it is unknown whether the absence of data in these fields is related to the lack of information or the non-manifested behavior.

**Table 4:** Percentages (%) of missing data for each field of the FATALITIES table per study area.

FFEM-DB fields (FATALITIES table)	BAL	CAT	CYP	CZE	GER	GRE	ISR	ITA	POR	SFR	TUR	UK	FFEM-DB
Missing data (%)													
Age	0	13	29	36	42	15	52	2	38	9	65	12	39
Gender	26	7	12	7	32	2	26	2	6	3	35	3	20
Residency	26	17	100	46	58	12	42	40	29	18	31	20	33
Victim’s condition	26	11	24	80	50	20	38	21	26	18	90	23	57
Victim’s activity	35	23	24	70	73	25	44	33	55	44	82	38	61
Accident place	26	6	24	32	36	6	30	4	12	7	64	12	36
Accident dynamic	30	3	35	49	45	3	28	4	16	24	20	13	20
Death cause	0	1	47	10	34	0	11	3	88	1	31	0	20
Total missing data (%), per study area	21	10	37	41	46	10	34	14	34	16	52	15	36

Among the evaluated fields, the lowest percentages of missing data correspond to gender (20%), accident dynamic (20%), and cause of death (20%), while the highest is associated with victim’s activity (61%). The study area of Turkey exhibits the highest proportion of missing data for the selected fields (52%), while those of Catalonia and Greece have the lowest (10%). Temporal change of field completeness

Table 5 shows the annual evolution of missing data (%) in the fields of the FATALITIES table for the total FFEM-DB area. Overall, there was a decreasing trend in missing data, which reduced from 47% in 1980 to 23% in 2020. The improvement of completeness over time reflects increased access to information, suggesting even better recording of such data in the future.

**Table 5:** Percentage (%) of missing data in the fields of the FATALITIES table for each study area and the total FFEM-DB area.

Missing values (%)	BAL	CAT	CYP	CZE	GER	GRE	ISR	ITA	POR	SFR	TUR	UK	FFEM-DB
1980						0	40			25	48		47
1981	0		75	25	100			10			51		47
1982		29		25				17			59	25	42
1983		0			100			13	59		64		60
1984		13	50	50				6					39
1985				38		0					50		38
1986		13		38	100			16		25	44		43
1987		13		43			19	13	0		43		31
1988		5		75	52	6				22	49		42
1989	75	0				0		4	17		54	25	38
1990	75	13		31	71	24	100	13	13	25	70		60
1991		19		13	25		29	11			54		40
1992				25			34	15		12	46		16
1993		38		25	73			17		22	54		29
1994		8	100	25	100	21	88	22	19	8	53	0	29
1995		13		31	60			14		0	71		65
1996		13	13	44		10	88	12	29	16	44		36
1997		63	88	55	6	13	38	19	30		41	13	43
1998		13		48	38			19		13	67	25	59
1999		0			33			14		15	43		22
2000		2		50	8	13		13	25	25		27	15
2001			25	13	0	3	75		34	29	40		35
2002				33	67			23		20	64		49
2003		0	13	38	8	7		9		33	70	0	31
2004		0						13	19	0	37	0	26
2005		4		38	27	13		11		16	51	8	31
2006	75	0	13	23	3		21		15	6	39		30
2007	75	13		13	13	0	25		38		41	23	26
2008					16	0		8	25	0	30	17	16
2009			25	51		2		21			38	2	33
2010				47	18	0	6	16	13	13	30	0	23
2011					25			15	25	9	44	13	25
2012		0		50	13	19		13		6	41	6	27
2013				39	28	6	21	10		0	23	13	19
2014		0			29	0		12		18	46		25
2015		0		38	13	6	0	6	38	15	49	0	23
2016		0			33	5	88	8			41	0	29
2017		0			50	18		11	25		39		20
2018	2	6	13	25	25	0	3	8	25	7	38	0	12
2019		8		13		0	25	8		1	70	0	18
2020	4	5		20		15	16	22		28	33	42	23

Note: empty cells denote years without FFs.

As the level of description of the FFs within FFEM-DB is very high, with 11 parameters required for the overall description of death conditions (FATALITIES table), missing values are expected. The percentage of missing values is quite large for some fields, especially at the beginning of the study period. Nevertheless, we consider all fields essential for analyzing the FF circumstances. Moreover, given the large sample, we do not consider that studies addressing the vulnerability of citizens to floods would be undermined. Regardless the acknowledged completeness trends, FFEM-DB creates an extensive dataset that allows study of flood mortality from multiple aspects (related to the different variables included), addressing the limitations mentioned in the introductory section of this study. Finally, we also expect more opportunities to fill these fields in the future through focused research and better information means.

External evaluation is based on the comparison with international databases and literature regarding the FFs coverage achieved. Finally, an evaluation through specific events that have been well-documented is performed.

### Overall coverage

Comparative analysis and evaluation of FFEM-DB in relation to other disaster databases require the high spatial density of FFs data to be adapted to the spatial levels used within other study areas to ensure comparability between reported FEs among databases.

Four independent publicly accessible disaster impact databases were considered for the evaluation of FFEM-DB completeness as to the total number of FFs: the Emergency Events Database (EM-DAT)^20^, the Dartmouth Flood Archive (DFA)^49^, the European Past Floods (EPF)^50^, and the Historical Analysis of Natural Hazards in Europe-HANZE-Events database (HANZE-E)^51^. The respective specifications are presented in online-only Table 2.

When comparing FFEM-DB with these databases, the following issues should, however, be considered:

- The external databases used for the comparison focus on catastrophic events irrespective of the occurrence of FFs, while the FFEM-DB focuses only on fatal events regardless of the overall induced societal impact of each case. Also, the information that the aforementioned databases provide about FFs is limited to the number of deaths, with no reference to the circumstances of each death.
- Each of the external databases refers to a different study period. EM-DAT is the only one covering the entire 1980–2020 period, which coincides with the FFEM-DB study period. DFA starts later (in 1985), while EPF and HANZ-E finish earlier (in 2015 and 2016, respectively).
- The external databases report on different geographical coverage and administrative level resolutions. This characteristic affected the comparison for the FEs in BAL, CAT, and SFR as data are not available at this administrative level in the other four impact databases, although basic information on the regions that the event affected within a country is always reported. An analysis of the FEs and associated FFs for these study areas is possible with EM-DAT and HANZE-E, after a thorough study of the reported events in Spain and France.

**Fig. 4:**
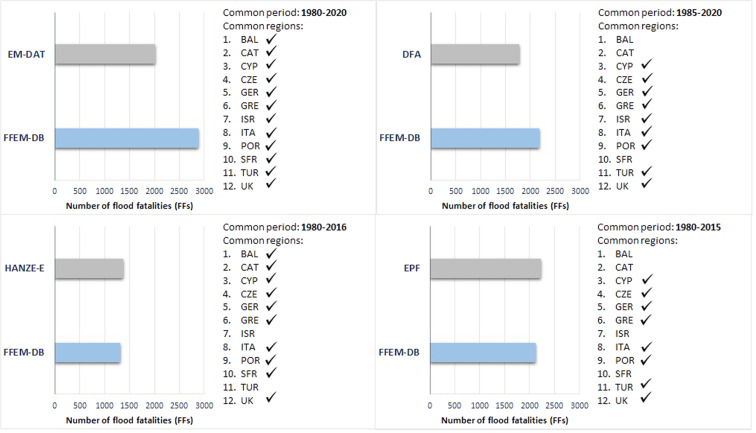
Number of flood fatalities (FFs) in FFEM-DB and the respective estimates of the four disaster databases (EM-DAT, DFA, EPF, and HANZE-E).

Figure 4 shows the number of FFs in FFEM-DB and the respective estimates of the four disaster databases. Only FFs corresponding to common periods and territories are considered in each case.

As demonstrated in online-only Table 2, the disaster databases also include fatalities from phenomena other than floods but related to them, such as landslides. However, it should be noted that where possible, based on analysis of published articles, extreme landslide events included in disaster databases have been excluded from the list of the events used in the comparative analysis. For example, for ITA, three extreme landslide events were excluded, namely the Cavalese-Stava mudflow in July 1985 that caused 329 fatalities^52^, the Giampilieri landslides in October 2009 that caused 37 fatalities^53^, and the landslide in May 1998 in Southern Italy^54^ that caused 148 fatalities.

Apart from the landslide fatalities, FFEM-DB did not consider FFs resulting from storm surge, coastal water, and infrastructure failure, such as dam break. For all the above reasons, the numbers of fatalities shown in Fig. 4 are not entirely comparable. However, the results indicate the level of FFs coverage reported by FFEM-DB. In particular, FFEM-DB contains 48% and 22% more FFs than EM-DAT and DFA, respectively, which is most likely related to the non-inclusion by the latter of small-scale fatal floods. The lower number of FFEM-DB FFs compared to EPF (−5%) and HANZE-E (−4%) is a result of the inclusion of losses of life from other phenomena (e.g., rain-induced landslides), but which cannot be easily distinguished. It has to be noted that currently, FFEM-DB is available for a more extended period than HANZE-E and EPF and more territories (i.e., Turkey, Israel).

### High-impact events coverage

Table 6 presents the results for the number of high-impact FEs and associated FFs per study area, derived by the two databases, FFEM-DB and EM-DAT. The EM-DAT was selected for this comparative analysis, as it covers the whole study period and geographical area of the FFEM-DB. Choosing events with 10 or more FFs eliminates the threshold bias associated with EM-DAT’s mandatory event entry criteria. The events that were specified in EM-DAT as landslides, dam breaks, or storm surge events were excluded from the comparison. Out of the 48 flood events recorded within EM-DAT (with 10 or more FFs), which concern the examined areas, 11 (23%) were excluded for the reasons mentioned above.

**Table 6:** Number of high-impact FEs (with 10 or more FFs) and associated FFs in FFEM-DB and EM-DAT, for the 1980-2020 period.

Study area	Number of FEs with 10 or more FFs		Number of associated FFs		Difference % of FFEM-DB FFs from EM-DAT FFs
	FFEM-DB	EM-DAT	FFEM-DB	EM-DAT	
BAL	1	1	13	13	0
CAT	1	0	12	0	100
CYP	0	0	0	0	—
CZE	3	3	63	60	5
GER	1	1	22	27	−19
GRE	2	1	37	11	236
ISR	1	2	14	27	−48
ITA	7	5	123	137	−10
POR	2	1	29	19	53
SFR	8	8	192	201	−4
TUR	28	17	706	533	32
UK	0	0	0	0	—
Total (1980–2020)	54	39	1211	1028	18

Overall, FFEM-DB contains 18% more FFs than EM-DAT for the FEs with 10 or more FFs. In particular, at the study area level, the comparison reveals different FF numbers for nine out of the 12 study areas. FFEM-DB includes more FFs for CAT, CZE, GRE, POR, and TUR, and less for GER, ISR, ITA, and SFR than EM-DAT. For CYP and UK, there were no FEs with more than 10 FFs; thus, they are not included in the comparison.

In the EM-DAT database, the affected Spanish regions are mentioned descriptively in each event, so it is possible to export events by region. Therefore, CAT was found to be included among other regions for two events with more than 10 FFs in June 2000 and October 2018. However, according to the literature, the June 2000 event caused five deaths in CAT^55^, thus it was excluded from comparison for CAT. In the October 2018 event, all the FFs took place in Mallorca (BAL)^56^. In addition, CAT was not included in the affected areas of EM-DAT in the November 1982 event, in which 14 FFs were recorded in CAT as reported by FFEM-DB and documented in relevant scientific articles^57,58^.

For CZE, both databases include three FEs that took place during the period under review, with FFEM-DB having 5% more FFs^59,60^.

For GER, only one FE is reported by both databases; however, the number of FF differs, 27 in EM-DAT compared to 22 in FFEM-DB (22). Careful analysis of the literature^61,62^ indicates that 20 FFs occurred, so the additional FFs in the EM-DAT are likely to reflect fatalities arising from other hazards.

For GRE, FFEM-DB includes more high-impact events, resulting in a higher number of FFs by a factor of two. The Greek FEs and FFs included in FFEM-DB have been validated through scientific publications focusing on the analysis of FFs in the country^16,63^.

For ISR, EM-DAT includes one more high-impact FE in October 1997, when most of the 13 fatalities occurred in car accidents caused by hazardous driving conditions resulting from heavy rainfall, as reported by local media^64^. According to the sources of FFEM-DB, only four FFs resulted from flooding, and therefore this FE is considered to have less than 10 FFs and is excluded from the comparison.

For ITA, FFEM-DB includes more high-impact FEs (seven) than EM-DAT (five), but a lower by 10% number of FFs. Inconsistencies were, however, found regarding the number of FFs provided by EM-DAT for some FEs. In the September 2000 flood event, EM-DAT reported 16 FFs in Soverato, Calabria, while the exact number of FFs was 13^65^. In addition, the November 1994 event is classified as a river flood, when in fact, landslides occurred and were responsible for several deaths^66^. Finally, the Versilia event in 1996 caused many fatalities, and while almost all of which resulted from debris flows^67^, in EM-DAT they were attributed to floods.

For POR, it should be noted that the FFEM-DB only contains FEs and FFs for Portugal mainland, excluding Madeira and the Azores archipelagos. This is why the February 2010 landslide/flash flood event in Madeira^68^ was excluded from this comparison. Another two events in December 1981 and January 1996 were excluded from the comparison, as both caused deaths attributed to landslides^69,70^. Beyond that, FFEM-DB includes two high-impact FEs, with only one of them listed in EM-DAT. The number of FFs for this event is comparable among the two databases.

For SFR, FFEM-DB and EM-DAT include the same FEs, while FFEM-DB reports nine fewer FFs (−4%) than EM-DAT.

For TUR, FFEM-DB includes 29% more FFs than EM-DAT. Out of the 28 Turkish high-impact FEs included in FFEM-DB, only 13 (46%) are also reported in EM-DAT, which, however, includes four events of which the number of FFs in FFEM-DB is marginally less than 10. All TUR high-impact FEs have been cross-referenced with local databases^71,72^.

### Accuracy evaluation through specific events

To evaluate further the accuracy of FFEM-DB, the number of FFs for specific well-documented flood events was compared against those reported in international databases. For the validation, the actual number of fatalities was derived from scientific publications and/or governmental reports describing these well-known events. In online-only Table 3, we present the comparison and relevant documentation of all the notable flood events that occurred in the FFEM-DB area and study period causing more than 15 FFs, showing that FFEM-DB has more accurate values than other existing databases with regard to the number of FFs.

Comparisons with international databases showed that the FFEM-DB achieves high coverage of FFs caused by flash floods, urban floods, and river floods while giving special attention to the quality, immediacy, and reliability of the sources it draws the information from. This is supported by the variety of sources used in each country/region included in FFEM-DB and the local character of information. The closeness of the information source to the actual flood events and the cross-checking between different sources enhance the completeness, accuracy, and reliability of the overall dataset.

## Usage Notes

The FFEM-DB database can be easily accessed and downloaded from the 4TU Centre for Research Data^39^, and data can be directly used for analysis (10.4121/14754999.v2). This database is intended to act as a large pool of publically accessible data for analysis of death circumstances over territories within the Euro-Mediterranean region. The relational structure of the database allows for analyses at the FF, study area, country, territorial, and NUTS levels. The FATALITIES table provides granular data on the FF profiles and circumstances. Each FF is further linked to geographical data (LOCATION table) and demographics at the NUTS levels (NUTS 3 table).

The data and their structure allow easy integration into databases intended to assess and analyze the societal impacts of disasters related to weather and climate. To this end, we provide, in the following, specific examples of analyses that can be applied. The extensive geographical area of the FFEM-DB dataset offers the opportunity to:

- Compare flood mortality in different geomorphological settings (e.g., flat areas of Germany or the Czech Republic against the high-inclination areas of Greece or Italy), as well as in different landcover and urbanization settings.
- Compare flood mortality and death circumstances among areas with different policies and measures aiming to address flood risk mitigation, such as through driving education, road network management, risk signage, and the adaptation of impact-based warnings.
- Examine the impact of risk mitigation policies and initiatives on flood mortality. For example, our dataset can be used to compare an area/country that uses a “turn around don’t drown” – type of awareness campaign^48^ with an area/country that does not, in terms of vehicle-related flood fatalities, as a complementary criterion on the efficiency of such campaigns.

## Data Availability

FFEM-DB is available in the 4TU Centre for Research Data^39^, 10.4121/14754999.v2). It includes the following files: a) a comma-separated values (csv) file, named “Fatalities.csv” that contains the structure and data of the FATALITIES Table (Table 3); b) a comma-separated values (csv) file, named “Location.csv” that contains the structure and data of the LOCATION Table (Table 2); c) a comma-separated values (csv) file, named “NUTS 3.csv” that contains the structure and data of the NUTS 3 Table (Table 2); d) the readme (txt) file, containing the description of the database structure.

## Online-only Tables

**Online-only Table 1:** Sources of flood events (FEs), flood fatalities (FFs), and relevant information included in FFEM-DB.

Study area	Coverage	References/Cstslogs	Description	Sources	Comments
Balearic Islands (BAL)	1403–2010	InunIB, Grimalt and Rosselló^30^	A record of FFs and their characteristics as part of a catalog of historical high-impact FEs.	Hard-copy & digital archives of regional newspapers for the 20th and 21st centuries. Chronicles and historical records for the 15th to 19th centuries. Available at:	The database is exhaustive, covering all the flood events that cause impact in the Balearic Islands.
				10.48088/ejg.m.gri.11.3.6.21	
	2011-today	InunIB 2.0	A record of FFs and characteristics.	Hard-copy & digital archives of regional newspapers; local online news pages; local and regional TV news coverage. Field research.	
	1960–2018	Fatalities DB, Grimalt, Rosselló and Bauzà^56^	List of deadly flood events in Mallorca. Analysis of flood-related mortality.	Hard-copy & digital archives of regional newspapers combined with field research. 10.1111/jfr3.12644	Exhaustive research of all the flood-related victims.
Catalonia (CAT)	1900-today	INUNGAMA, Llasat *et al*.^58,73^	INUNGAMA: Flood events database in Catalonia classified according to their severity.	INUNGAMA: Impact information from insurance companies (e.g., Insurance Compensation Consortium), official government or municipalities reports, scientific and technical studies, newspapers, Internet contents, social networks, and citizen contributions (Floodup App). This database also includes hydrometeorological information.	INUNGAMA: The database covers the flood events produced in Catalonia from 1900.
		PRESSGAMA, Llasat *et al*.^24^	PRESSGAMA is a database that contains selected and classified information from all the news on natural hazards and climate change published in La Vanguardia newspaper since 1981.		
Cyprus (CYP)	1980-today	Preliminary flood risk assessment report of Water Development Department	A record of FFs, their profiles, and death circumstances as part of a catalog of historical high-impact FEs.	Multiple sources, including reports of the Cyprus Fire Service, the Department of Meteorology of Cyprus, the Water Development Department and the Hydrology Service of the Ministry of Agriculture Rural Development and the Environment, as well as national newspapers (e.g. Eleftheria, Cyprus, Salpix, Evagoras, Alitheia, Enosis, Simerini, Politis and others).	The catalog of FFs is exhaustive.
Czech Republic (CZE)	1961-today	Historical-climatological database of the Department of Geography, Masaryk University	A record of FFs, their profiles, and death circumstances.	The database consists mainly of published newspapers and their internet versions complemented by other documentary sources (chronicles, publications, policy and statistical records, etc.).	The database is exhaustive, covering fatalities and injured for all the weather-related events.
Germany (GER)	1980-today	Not published	A record of FFs, their profiles, and death circumstances.	A newspaper aggregator database was used (genius.de). It comprises 190 million news articles since 1980, published in more than 300 different news outlets, however sparse article numbers 1980-1995.	The catalog of FFs is systematic and was elaborated by using text-mining tools. Close reading was used to identify the death circumstances and profile of the victims.
	1980–1998	Not published	A record of FFs numbers.	Report: Munich Re (1999) Natur-katastrophen in Deutschland – Schadenerfahrungen und Schadenpotentiale (*Natural disasters in Germany - loss experience and loss potentials*). Munich Re.	The report includes the number of fatalities per event with no additional information regarding the profiles or death circumstances.
Greece (GRE)	2000-today	Papagiannaki *et al*.^28^	A systematically updated & online available database of damaging weather-related events.	Hardcopy & digital archives of 'Ethnos' and 'Rizospastis' national newspapers; local online news pages. The sources of each record are available in the online version of the database (https://www.meteo.gr/weather_cases.cfm).	The database is exhaustive, covering all the weather-related events that cause societal impacts in Greece.
	1980-today	IERSD/NOA catalogs	A record of FFs, their profiles, and death circumstances. A record of historical high-impact FEs.	Hardcopy & digital archives of 'Ethnos' and 'Rizospastis' national newspapers; monthly bulletins redacted at the	The catalog of FFs is exhaustive. The catalog of high-impact FEs for the period 1980-2000 includes all the fatal events.
				National Observatory of Athens (https://www.meteo.gr/Monthly_Bulletins.cfm); local online news pages.	
Israel (ISR)	1948-2021	Dataset of the University of Haifa Inbar^74^	It includes climatological and hydrological characteristics of the flood and details on fatalities	Newspapers and data by government authorities (Hydrological and Meteorological services)	Catalog of flood events and other hazards accompanied with fatalities
Italy (ITA)	1980-today	Salvati *et al*.^75^	Gender, age and circumstances analysis of flood and landslide fatalities in Italy.	Multiple sources, including official government reports and documents, online newspaper articles, hard copy and digital national and regional newspaper archives, websites, written reports and interviews to eyewitnesses, survivors’ inquiries, and petitions.	The catalog of landside and flood events with human consequences includes information on the dead, injured people, missing persons, evacuated, and homeless people from 589 AD to 2020. The recent portion of the catalog (1970-2020) is considered quite complete, including almost all the fatal events by type of processes, from very low intensity (causing one fatality) to the destructive ones.
Portugal (POR)	1980-today	Disaster database	A systematically updated & online available database of flood and landslide cases that caused human damages.	Hardcopy and digital archives of the newpapers “Diário de Notícias”, “Jornal de Notícias”, “Correio da Manhã”, “O Público”, “Diário do Alentejo”, “Jornal do Fundão”, “Região de Leiria” and “O Algarve independente”.	The database is exhaustive, covering all flood and landslide events that caused societal impacts in Portugal for a longer period (1865-2020).
		Zêzere *et al*.^69^	A record of FFs, their profiles, and death circumstances.		
		Pereira *et al*.^29^			
		Pereira *et al*.^25^			
Southern France (SFR)	1800–2000	Antoine *et al*.^76^	List of deadly flood events in Languedoc-Roussillon (4 departments)	Scientific paper available online https://www.jstor.org/stable/23456069?seq=1#metadata_info_tab_contents	
	1980–1995	Geosciences consultants^77^	List of deadly flood events in France. Analysis of flood-related mortality	Hard copy report	
	1988–2011	Boissier^78^	Ph.D. on flood-related fatalities in Southern France. Available Online: https://tel.archives-ouvertes.fr/tel-00940888	Hardcopy and digital archives of the newspapers “Midi libre”, “La Provence” and other local newspapers verified on the field near municipalities and emergency services.	
	1980-2020	VICT-IN	A record of FFs, their profiles, and death circumstances.	University of Paul Valéry Montpellier 3 Lagam laboratory	
Turkey (TUR)	1980–2007	Not published	Severe weather records from newspaper archive	Milliyet Newspaper Archive http://gazetearsivi.milliyet.com.tr	The electronic archives of Milliyet and Cumhuriyet are searched for flood-relevant words, and events are identified by going through the details the newspaper articles covered.
	1980–2020	Not published	Severe weather records from newspaper archive	Cumhuriyet Newspaper Archive https://egazete.cumhuriyet.com.tr	
	2000-2020	Not published	Severe weather records found online and validated via cross-checks	Various online newspapers	
	1997–2020	Not published	Severe weather records from meteorological stations	Various online newspapers	Extracted from hard copies of severe weather records
United Kingdom (UK)	1980-present	Not published	A new database was compiled of FF information from around the UK for this project based on reported accounts.	Hard copies and digital newspaper archives were accessed to determine details, coupled with the BBC website (digital) archived stories to compile the database. A rich history of newspaper records in the UK offers considerable opportunities for a long record of FFs to be constructed for the UK back to the 1800s.	All records are extracted from newspaper accounts. The total number of FFs is comparable with contemporary accounts and those subsequently reported in technical reports for particular storms, e.g., https://researchbriefings.files.parliament.uk/documents/CBP-7424/CBP-7424.pdf

**Online-only Table 2:** Specifications of the comparative databases: EM-DAT, DFA, EPF, HANZE-E, and FFEM-DB.

	EM-DAT Emergency Events Database	DFA	EPF	HANZE-E	FFEM-DB
		Dartmouth Flood Archive	European Past Floods	Historical Analysis of Natural Hazards in Europe	Database of Flood Fatalities from the Euro-Mediterranean region
Scale	Global	Global	EU Member States (includes TUR)	EU Member States	Study areas in Fig. 2
Period	Since 1900^1^	Since 1985^2^	1980–2015^3^	1870–2016^4^	1980–2020
Grid	Country, region, basin, lat., long.	Country, provinces, towns, cities	EU units of management	Country NUTS 3, 2010 edition	Country, NUTS 2 and 3, 2020 edition
Database producer	Centre for Research on Epidemiology of Disasters	Dartmouth Flood Observatory	European Environment Agency	(TUDelft)^51^	The authors of the present paper
Link	www.emdat.be/	www.dartmouth.edu/floods/Archives/	www.eea.europa.eu/data-and-maps/data/european-past-floods	10.4121/uuid:62d3fc79-6638-480f-8d64-9c8d200bd41c	10.4121/14754999.v2
Focus of database	Natural and technological disasters	Floods	Floods	Floods	Flood fatalities
Inclusion criteria	• 10 or more people killed	• Significant damage to structures or agriculture	• Floods reported by EU Member States for the EU Floods Directive (2007/60/EC)	• At least one of the four statistics (area flooded, deaths, persons affected, the monetary value of losses) is available for a given event. However, if no persons were known to have been killed or missing, at least one of the other statistics had to be available.	• FFs due to river floods and flash floods
	• 100 or more people affected	• Decades intervals since the last similar event	• Data provided by relevant national authorities	• Insignificant floods, i.e., events that affected only a small part of one region, with no fatalities and less than 200 persons affected, were not included.	• FFs due to storm surges and dam breaks are not included
	• declaration of a state of emergency	• and/or fatalities	• Data from EM-DAT and DFA	• Availability of information on the date, regions affected, type, and cause of the flood	
	• and/or a call for international assistance				
Data on FFs	Number of fatalities per event	Number of fatalities per event	Number of fatalities per event	Number of fatalities per event	Several parameters for each fatality

^1^EM-DAT: file emdat_public_2021_01_04_query_uid-aPsirp, accessed 04-01-2021.

^2^DFA: file MASTERLIST, accessed 04-01-2021.

^3^EPF: file FloodPhenomena_2015_public, accessed 02-01-2021.

^4^HANZE-E: HANZE Events_floods, accessed 23-12-2020

**Online-only Table 3:** Example comparison of FFs records between FFEM-DB and international databases on specific multiple fatality and well-documented flood events.

Flood event	Number of FFs recorded					Number of FFs based on event-specific studies	Sources
	EM-DAT	EPF	DFA	HANZE-E	FFEM-DB		
Moravian-Silesian region, Czech Republic, June-July 2009	13	15	X	15	15	15	Řezáč *et al*.^79^
Germany, August 2002	27	27	55	27	22	21	Kreibich and Merz^80^
Mandra, Greece, Νovember 2017	23	N/R	16	N/R	24	24	Diakakis *et al*.^81^, Diakakis *et al*.^16^
							Inspector General of Public Administration^82^
Sardinia, Italy, November 2013	18	18	18	18	18	18	Cossu *et al*.^83^, Righini *et al*.^84^
Piedmont, Italy, November 1994	68	68	83	68	46	44	Regione Piemonte^85^, Luino and Padano^66^
Lisbon Metropolitan Area, Portugal, 18-19 November 1983	19	19	N/R	18	18	18	Pereira *et al*.^70^, Trigo *et al*.^86^
South East France, October 2020	26	N/R	X	N/R	18	15	Prakash and Manconi^87^
South-western France, October 2018	14	N/R	X	N/R	15	15	Caumont *et al*.^88^
South East France October 2015	20	X	16	20	20	20	Saint Martin *et al*.^89^
Var region, France, June 2010	25	25	19	26	26	26	Vinet *et al*.^9^
Gard region, France, September 2002	23	23	23	23	24	24	Delrieu *et al*.^4^, Ruin *et al*.^90^
Aude region, France, November 1999	36	36	27	35	29	30	Vinet *et al*.^45^, Gaume *et al*.^3^
Vaison-la-Romaine, France, September 1992	47	41	38	46	49	41-42	Vinet^91^, Anisimov^92^, Gibert and Gouy^93^
Southeastern Anatolia, Turkey, October 2010	X	1	X	N/R	44	41-46	Koç and Thieken^71^, Turkoglu^94^
Istanbul (Marmara), Turkey, September 2009	40	40	31	N/R	39	31–32	Koç and Thieken^71^, Komuscu and Celik^95^, Turoglu^96^
Sanliurfa, Diyarbakir, Sirnak, Batman (Southeastern Anatolia), Turkey, October-November 2006	47	58	46	N/R	44	47	Koç and Thieken^71^
Various locations (Black Sea area, Central Anatolia and others), Turkey, July 2002	34	36	34	N/R	39	33–40	Koç and Thieken^71^, Ceylan^97^
Trabzon, Eastern Black Sea, Turkey, Aug-98	60	60	50	N/R	47	47–50	Yuksek *et al*.^98^, Sahin^99^
Zonguldak, Karabuk and others, Turkey, May 1998	10	14	19	N/R	17	10-27	Koç and Thieken^71^
Izmir, Antalya and others, Turkey, November 1995	63	61	62	N/R	61	61	Koç and Thieken^71^, Komuscu and Celik^95^
Isparta, Turkey, July 1995	X	74	X	N/R	75	74–75	Ozden^100^, Koç and Thieken^71^
Diyarbakir, Malatya, Adiyaman, Elazig, (Eastern Anatolia), Turkey, May 1991	42	38	42	N/R	33	38	Koç and Thieken^71^
Giresun, Trabzon, Turkey, June 1990	51	51	48	N/R	61	51–57	Koç and Thieken^71^, Yuksek *et al*.^98^
Concordance between the number of FFs^1^							
Exact matches	6	10	5	5	14		
Approximate matches	6	3	6	2	5		
Wide differences	9	6	6	3	5		
Number of events included	21/23	19/23	17/23	10/23	23/23		

Note: X = Event is missing, N/R = Not reported due to an uncovered region or period. For certain events included in FFEM-DB or other databases, no individual studies were found for comparison, including Turkey July 1991, Turkey March 1980, Turkey July 1983, Turkey December 1981, and Turkey August 2010.

^1^Compared to ‘Number of FFs based on event-specific studies’. Events that are not geographically or temporally covered by the database are not considered. It is considered an exact match if the number is within a referenced range. If the difference is less than 10%, the number is considered an approximate match.

## Code availability

No custom code was used to generate or process the data described in the manuscript.
